# Lectures on Orthopædic Surgery

**Published:** 1864-04

**Authors:** 


Lectures on Orthopædic Surgery. Delivered at the Brooklyn Medical and Surgical Institute, with numerous Illustrations. By Louis Bauer, M.D., M.R.C.S., Eng.; Prof. of Anatomy and Clinical Surgery; Licentiate of the New York State Medical Society; Member of the New York Pathological Society; of the American Medical Association; Corresponding Fellow of the London Medical Society; Health Officer of the City of Brooklyn, &c. Reprinted from the Philadelphia Medical and Surgical Reporter. Philadelphia: Lindsay & Blakiston. 1864.

This is a monograph of 108 large, double column pages, written in the style of lectures. It is well illustrated by cuts showing the various deformities and the apparatus required in their treatment. The practical surgeon will find it an interesting and valuable work. For sale by S. C. Griggs & Co., booksellers, Lake Street, Chicago.

